# Supplementary figures and images for: Risk factor analysis and creation of an externally-validated prediction model for perioperative stroke following non-cardiac surgery: A multi-center retrospective and modeling study
Source: PLoS Med. 2025 Mar 21;22(3):e1004539. doi: 10.1371/journal.pmed.1004539 (PMC11927879; doi:10.1371/journal.pmed.1004539)

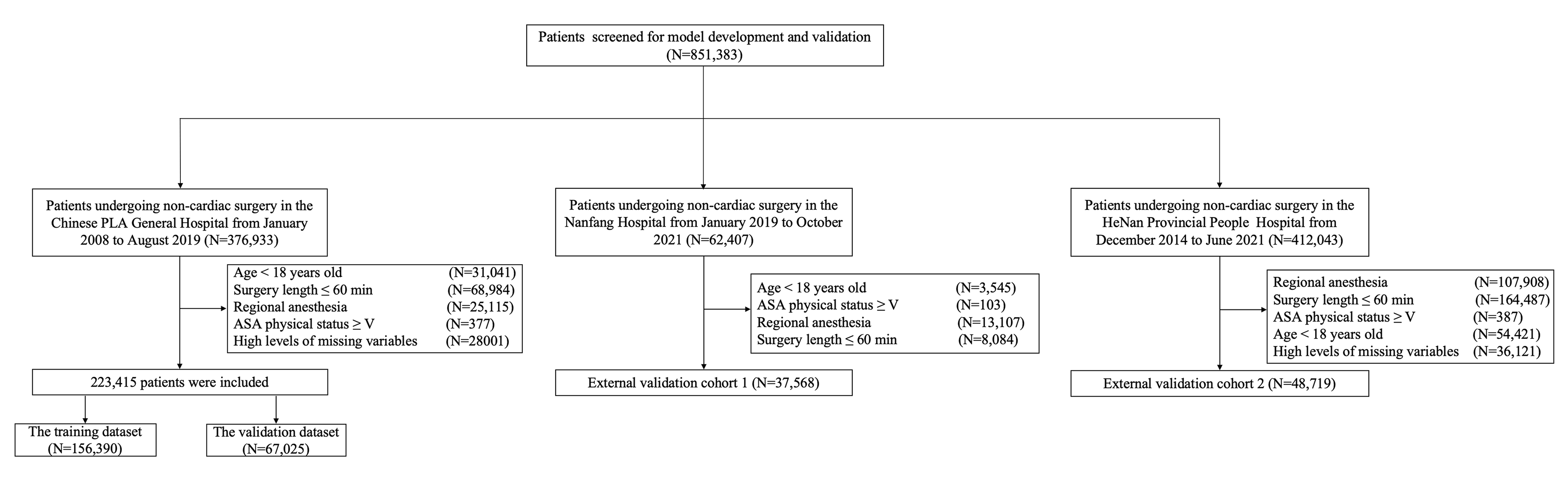

Supplement: S1 Fig — ASA, American Society of Anesthesiologists. (TIF) [file pmed.1004539.s001.tif]

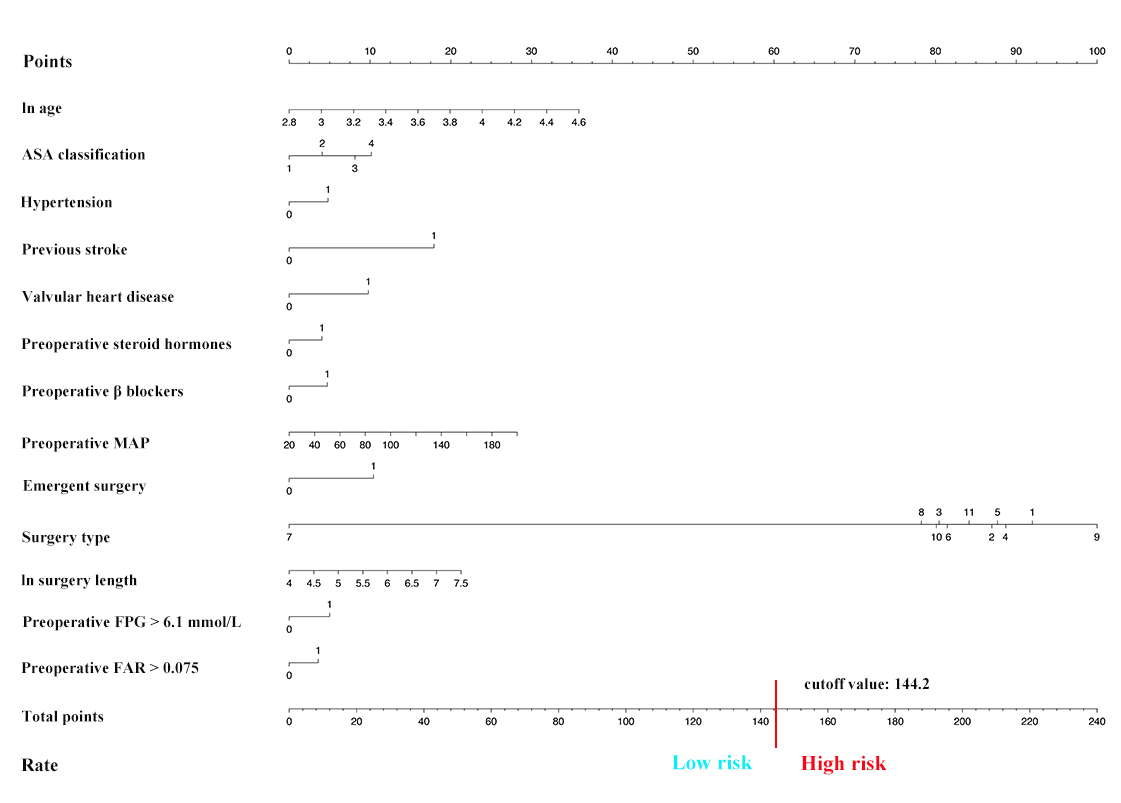

Supplement: S2 Fig — Age and surgery length were ln transformed. FPG and FAR were transformed to binary data according to the cut-off values. For binary data, 0 represents “no” and 1 represents “yes”. ASA, American Society of Anesthesiologists; MAP, mean arterial pressure; FAR, fibrinogen to albumin ratio; FPG, fasting plasma glucose. Surgery type 1, ear, nose, and throat; 2, obstetrics and gynecology; 3, abdominal surgery; 4, orthopedics; 5, stomatology; 6, urology; 7, general surgery; 8, other surgeries; 9, neurosurgery; 10, thoracic surgery; 11, vascular surgery. (TIF) [file pmed.1004539.s002.tif]

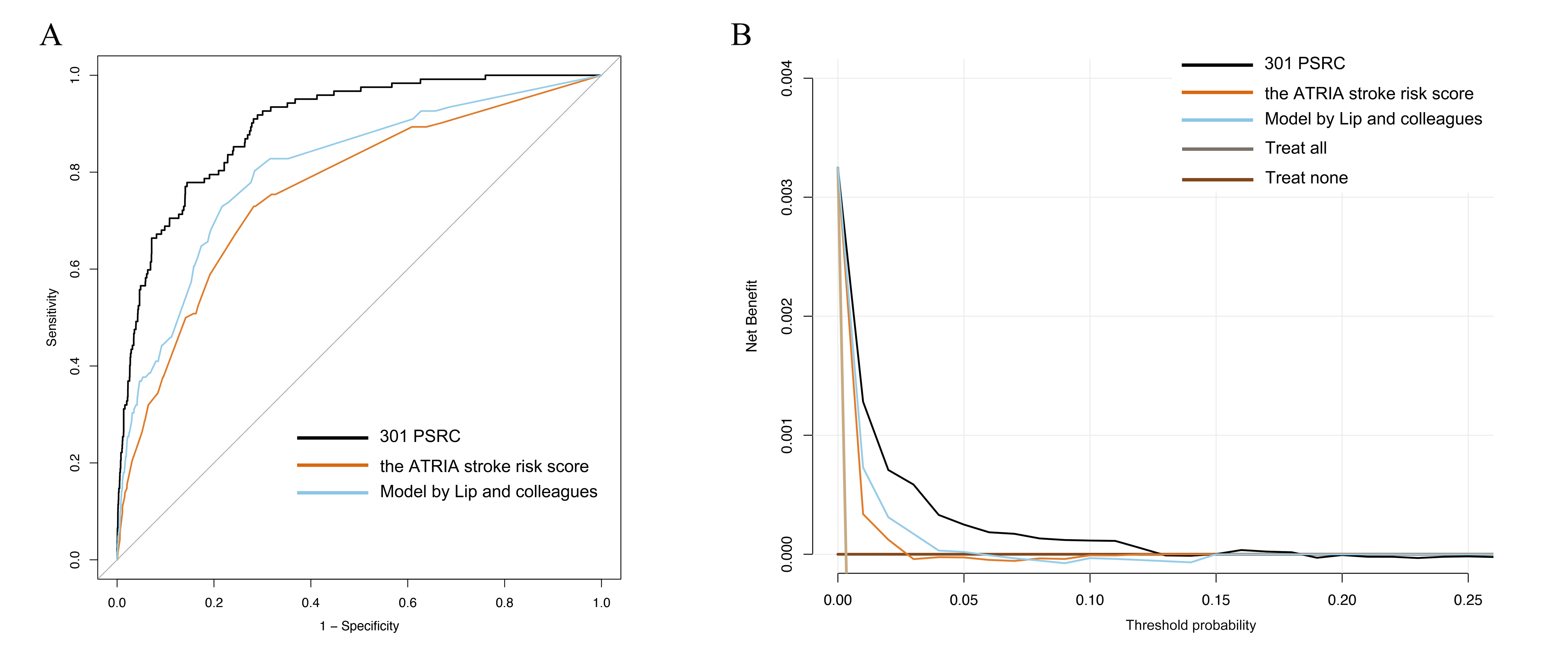

Supplement: S3 Fig — (A) The 301 PSRC showed higher AUC when compared to the model reported by Lip [15] and the ATRIA stroke risk score [14]. (B) The 301 PSRC exhibited a positive net benefit superior over the model reported by Lip and the ATRIA stroke risk score. (TIF) [file pmed.1004539.s003.tif]

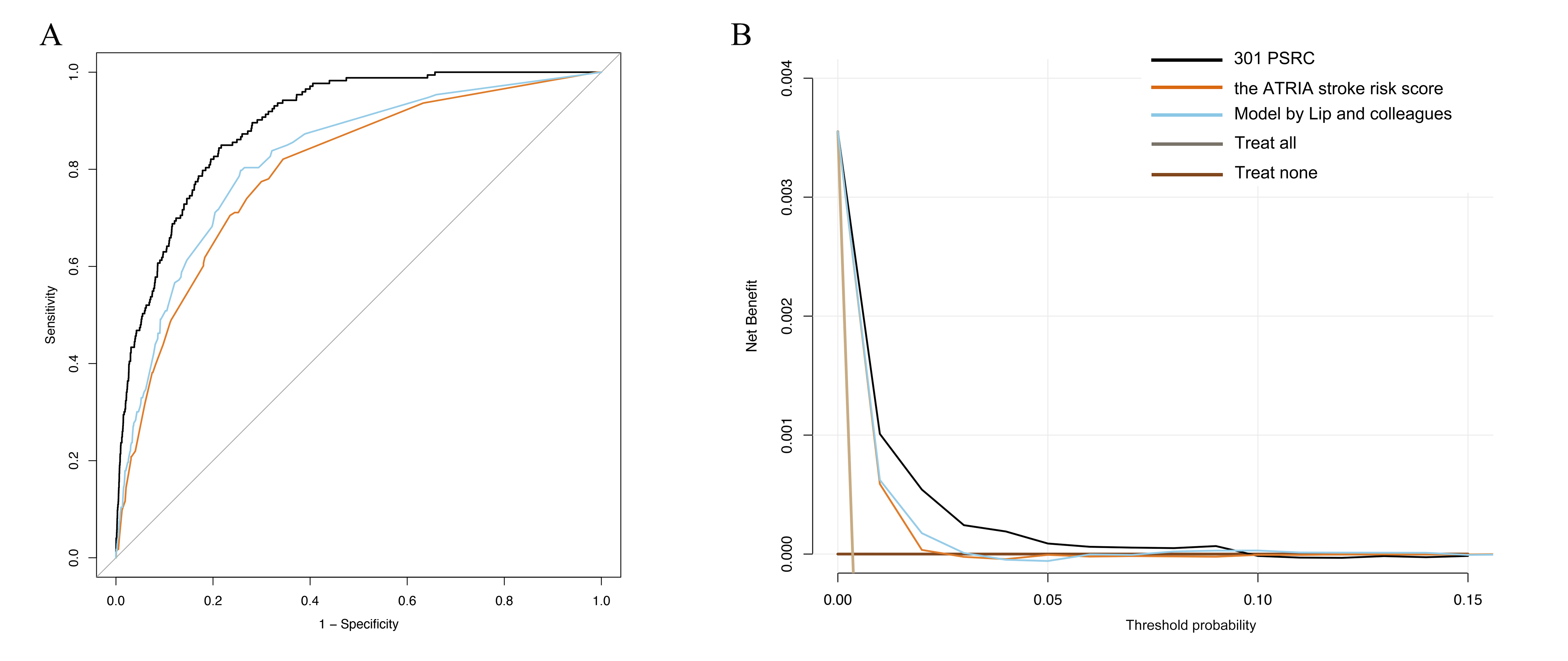

Supplement: S4 Fig — (A) The 301 PSRC showed higher AUC when compared to the model reported by Lip [15] and the ATRIA stroke risk score [14]. (B) The 301 PSRC exhibited a positive net benefit superior over the model reported by Lip and the ATRIA stroke risk score. (TIF) [file pmed.1004539.s004.tif]
