# Supplementary material for: Risk factor analysis and creation of an externally-validated prediction model for perioperative stroke following non-cardiac surgery: A multi-center retrospective and modeling study
Source: PLoS Med. 2025 Mar 21;22(3):e1004539. doi: 10.1371/journal.pmed.1004539 (PMC11927879; doi:10.1371/journal.pmed.1004539)
Supplement: S2 Table — (DOCX) [file pmed.1004539.s006.docx]

**Supplementary Table 2 ICD-9/10 Diagnosis Codes for Ischemic Stroke**

| Ischemic stroke | **ICD-9/ICD-10** | 433.X1/I63.X | Occlusion and stenosis of precerebral arteries with cerebral infarction |
| --- | --- | --- | --- |
|  | **ICD-9** | 434.X1 | Occlusion of cerebral arteries with cerebral infarction |
|  | **ICD-9/ICD-10** | 437.1/I67.81,  I67.89 | Other generalized ischemic cerebrovascular disease |
|  | **ICD-9/ICD-10** | 437.9/I67.9 | Unspecified cerebrovascular disease |

ICD, International Classification of Diseases.
