# Supplementary material for: Risk factor analysis and creation of an externally-validated prediction model for perioperative stroke following non-cardiac surgery: A multi-center retrospective and modeling study
Source: PLoS Med. 2025 Mar 21;22(3):e1004539. doi: 10.1371/journal.pmed.1004539 (PMC11927879; doi:10.1371/journal.pmed.1004539)
Supplement: S3 Table — (DOCX) [file pmed.1004539.s007.docx]

**Supplementary Table 3 Patient characteristics in the validation cohorts.**

| **Characteristics** | Nanfang Hospital | | | Henan Provincial People’s Hospital | | |
| --- | --- | --- | --- | --- | --- | --- |
|  | No postoperative ischemic stroke | Postoperative ischemic stroke | Total | No postoperative ischemic stroke | Postoperative ischemic stroke | Total |
|  | n = 37446 | n = 122 | n = 37568 | n = 48546 | n = 173 | n = 48719 |
| **Patient characteristics** |  |  |  |  |  |  |
| **Age, years** | 51 (39, 61) | 62.5 (51, 70) | 51 (39, 61) | 53 (41, 64) | 69 (63, 76) | 53 (41, 64) |
| **Sex** |  |  |  |  |  |  |
| male | 18289 (48.8) | 80 (65.6) | 18369 (48.9) | 23290 (48) | 103 (59.5) | 23393 (48) |
| female | 19157 (51.2) | 42 (34.4) | 19199 (51.1) | 25256 (52) | 70 (40.5) | 25326 (52) |
| **ASA classification** |  |  |  |  |  |  |
| Ⅰ | 9852 (26.3) | 8 (6.6) | 9860 (26.2) | 4020 (8.3) | 0 | 4020 (8.3) |
| Ⅱ | 24231 (64.7) | 57 (46.7) | 24288 (64.7) | 36024 (74.2) | 68 (39.3) | 36092 (74.1) |
| Ⅲ | 3053 (8.2) | 42 (34.4) | 3095 (8.2) | 7781 (16) | 105 (60.7) | 7886 (16.2) |
| Ⅳ | 310 (0.8) | 15 (12.3) | 325 (0.9) | 721 (1.5) | 0 | 721 (1.5) |
| **BMI, kg/m^2^** | 23.1 (20.8, 25.4) | 23.9 (21.0, 26.2) | 23.1 (20.8, 25.4) | 23.44 (21, 26.4) | 23.4 (20.2, 26) | 23.4 (21, 26.4) |
| **Hypertension, n (%)** | 8124 (21.7) | 69 (56.6) | 8193 (21.8) | 6688 (13.8) | 56 (32.4) | 6744 (13.8) |
| **Diabetes, n (%)** | 5310 (14.2) | 28 (23) | 5338 (14.2) | 4078 (8.4) | 39 (22.5) | 4117 (8.5) |
| **Myocardial infarction，n (%)** | 85 (0.2) | 0 | 85 (0.2) | 247 (0.5) | 5 (2.9) | 252 (0.5) |
| **Coronary heart disease，n (%)** | 1805 (4.8) | 10 (8.2) | 1815 (4.8) | 2155 (4.4) | 21 (12.1) | 2176 (4.5) |
| **Heart failure, n (%)** | 65 (0.2) | 1 (0.8) | 66 (0.2) | 92 (0.2) | 3 (1.7) | 95 (0.2) |
| **Atrial fibrillation, n (%)** | 151 (0.4) | 2 (1.6) | 153 (0.4) | 236 (0.5) | 6 (3.5) | 242 (0.5) |
| **Previous stroke, n (%)** | 984 (2.6) | 33 (27) | 1017 (2.7) | 2256 (4.6) | 53 (30.6) | 2309 (4.7) |
| **Valvular heart disease, n (%)** | 554 (1.5) | 3 (2.5) | 557 (1.5) | 390 (0.8) | 4 (2.3) | 394 (0.8) |
| **Angina pectoris, n (%)** | 52 (0.1) | 1 (0.8) | 53 (0.1) | 82 (0.2) | 4 (2.3) | 86 (0.2) |
| **Peripheral vascular disease, n (%)** | 2289 (6.1) | 28 (23) | 2317 (6.2) | 3649 (7.5) | 25 (14.5) | 3674 (7.5) |
| **Renal insufficiency, n (%)** | 692 (1.8) | 6 (4.9) | 698 (1.9) | 400 (0.8) | 0 | 400 (0.8) |
| **Malignant tumor, n (%)** | 13918 (37.2) | 50 (41) | 13968 (37.2) | 6412 (13.2) | 14 (8.1) | 6426 (13.2) |
| **Preoperative Hemoglobin, g/L** | 130 (118, 143) | 123 (110, 139.8) | 130 (118, 142) | 126 (111, 140) | 119 (104, 131) | 126 (111, 140) |
| **Preoperative serum albumin, g/L** | 40.5 (37.7, 43.8) | 37.8 (34.1, 40.7) | 40.5 (37.7, 43.8) | 40.6 (36.7, 44.1) | 36.2 (32.5, 39.5) | 40.5 (36.7, 44.1) |
| **Preoperative total bilirubin, μmol/L** | 9.3 (6.9, 12.6) | 10.3 (6.7, 16.4) | 9.3 (6.9, 12.6) | 9.8 (7.1, 14) | 9.9 (6.9, 15.9) | 9.8 (7.1, 14) |
| **Preoperative FPG, mmol/L** | 4.97 (4.6, 5.5) | 5 (4.6, 6.2) | 4.97 (4.6, 5.5) | 5 (4.4, 6) | 5.68 (4.7, 7.6) | 5 (4.4, 6) |
| **Preoperative thrombin time, s** | 17.4 (16.8, 18.2) | 17.2 (16.3, 18) | 17.4 (16.8, 18.1) | 16.4 (15.4, 17.5) | 16.2 (15.4, 17.5) | 16.4 (15.4, 17.5) |
| **Preoperative ACEI drugs, n (%)** | 935 (2.5) | 7 (5.7) | 942 (2.5) | 3840 (7.9) | 28 (16.2) | 3868 (7.9) |
| **Preoperative ARB drugs, n (%)** | 2413 (6.4) | 25 (20.5) | 2438 (6.5) | 2441 (5) | 21 (12.1) | 2462 (5.1) |
| **Preoperative Steroids, n (%)** | 3089 (8.2) | 11 (9) | 3100 (8.3) | 11479 (23.6) | 45 (26) | 11524 (23.7) |
| **Preoperative β-blockers, n (%)** | 1585 (4.2) | 19 (15.6) | 1604 (4.3) | 44012 (90.7) | 168 (97.1) | 44180 (90.7) |
| **Preoperative Calcium channel blockers, n (%)** | 5504 (14.7) | 52 (42.6) | 5556 (14.8) | 7203 (14.8) | 76 (43.9) | 7279 (14.9) |
| **Preoperative MAP** | 77 (68.7, 88) | 83.5 (71.3, 94.8) | 77 (68.7, 88) | 92.7 (84.7, 101.7) | 95.7 (86.7, 106.7) | 92.7 (84.7, 101.7) |
| **Perioperative aspirin, n (%)** | 1080 (2.9) | 21 (17.2) | 1101 (2.9) | 3705 (7.6) | 55 (31.8) | 3760 (7.7) |
| **Perioperative nonsteroidal drugs, n (%)** | 3845 (10.3) | 33 (27) | 3878 (10.3) | 44012 (90.7) | 168 (97.1) | 44180 (90.7) |
| **Emergent surgery, n (%)** | 2171 (5.8) | 19 (15.6) | 2190 (5.8 | 8017 (16.5) | 54 (31.2) | 8071 (16.6) |
| **Surgery type, n (%)** |  |  |  |  |  |  |
| ENT | 2378 (6.4) | 2 (1.6) | 2380 (6.3) | 3506 (7.2) | 4 (2.3) | 3510 (7.2) |
| Obstetrics and gynecology | 3838 (10.2) | 2 (1.6) | 3840 (10.2) | 7627 (15.7) | 0 | 7627 (15.7) |
| Abdominal surgery | 3856 (10.3) | 33 (27) | 3889 (10.4) | 13027 (26.8) | 46 (26.6) | 13073 (26.8) |
| Orthopedics | 5789 (15.5) | 11 (9) | 5800 (15.4) | 8364 (17.2) | 36 (20.8) | 8400 (17.2) |
| Stomatology | 1102 (2.9) | 1 (0.8) | 1103 (2.9) | 711 (1.5) | 1 (0.6) | 712 (1.5) |
| Urology | 2935 (7.8) | 8 (6.6) | 2943 (7.8 | 5470 (11.3) | 14 (8.1) | 5484 (11.3) |
| General surgery | 8607 (23) | 4 (3.3) | 8611 (22.9) | 4767 (9.8) | 4 (2.3) | 4771 (9.8) |
| Other surgeries | 794 (2.1) | 1 (0.8) | 795 (2.1) | 2272 (4.7) | 38 (22) | 2310 (4.7) |
| Neurosurgery | 4397 (11.7) | 47 (38.5) | 4444 (11.8) | 1087 (2.2) | 18 (10.4) | 1105 (2.3) |
| Thoracic surgery | 3661 (9.8) | 10 (8.2) | 3671 (9.8) | 593 (1.2) | 1 (0.6) | 594 (1.2) |
| Vascular surgery | 89 (0.2) | 3 (2.5) | 92 (0.2) | 1122 (2.3) | 11 (6.4) | 1133 (2.3) |
| **Surgery length, min** | 135 (93, 201) | 197 (120.3, 331.3) | 135 (93, 201) | 145 (100, 220) | 170 (120, 255) | 145 (100, 220) |
| **Amount of blood loss, ml** | 50 (5, 100) | 100 (20, 300) | 50 (5, 100) | 50 (10, 200) | 100 (20, 200) | 50 (10, 200) |
| **Intraoperative steroids, n (%)** | 7290 (19.5) | 57 (46.7) | 7347 (19.6) | 9520 (19.6) | 73 (42.2) | 9593 (19.7) |
| **NLR** | 1.9 (1.4, 2.8) | 3.02 (2.1, 4.4) | 1.93 (1.4, 2.8 | 2.48 (1.7, 4.6) | 3.43 (2.1, 6.6) | 2.49 (1.7, 4.7) |
| **PLR** | 125.9 (96.2, 167.9) | 170.2 (113.6, 215) | 126 (96.3, 168.1) | 140.5 (104.5, 197.2) | 155.6 (110.1, 24) | 140.6 (104.5, 197.3) |
| **FAR** | 0.07 (0.06, 0.09) | 0.09 (0.07, 0.14) | 0.07 (0.06, 0.09) | 0.07 (0.06, 0.10) | 0.10 (0.08, 0.15) | 0.07 (0.06, 0.10) |
| **Blood product usage, n (%)** | 2999 (8) | 25 (20.5) | 3024 (8) | 6516 (13.4) | 34 (19.7) | 6550 (13.4) |
| **Crystals, mL/kg/h** | 7.3 (5.1, 10.4) | 6.6 (4.9, 9.3) | 7.3 (5.1, 10.4) | 9.8 (7.1, 13.2) | 9.2 (5.9, 12.5) | 9.8 (7.1, 13.2) |
| **Colloids, mL/kg/h** | 2.0 (0, 3.7) | 2.4 (0.5, 4.1) | 2.05 (0, 3.7) | 3.4(2.3, 5) | 3.2 (2.2, 4.2) | 3.4 (2.3, 5) |
| **Morphine equivalents, mg** | 333 (167, 333) | 267 (25, 417) | 333 (167, 333) | 90 (90, 90) | 90 (75, 90) | 90 (90, 90) |

*P*-values were determined using χ2 or Fisher’s exact tests for categorical variables and analysis of variance or Kruskal-Wallis tests for continuous variables. ACEIs, angiotensin-converting enzyme inhibitors; ARBs, angiotensin II receptor blockers; ASA, American Society of Anesthesiologists; BMI, body mass index; ENT, ear, nose and throat; FAR, fibrinogen to albumin ratio; FPG, fasting plasma glucose; MAP, mean arterial pressure; NLR, neutrophil-lymphocyte ratio; PLR, platelet-to-lymphocyte ratio.
