# Supplementary material for: Risk factor analysis and creation of an externally-validated prediction model for perioperative stroke following non-cardiac surgery: A multi-center retrospective and modeling study
Source: PLoS Med. 2025 Mar 21;22(3):e1004539. doi: 10.1371/journal.pmed.1004539 (PMC11927879; doi:10.1371/journal.pmed.1004539)
Supplement: S4 Table — (DOC) [file pmed.1004539.s008.doc]

**Supplementary Table 4 Univariate analysis**

| **Variables** | OR (95% CI) | *P* value |
| --- | --- | --- |
| **ln Age** | 23.499 (14.486, 38.844) | < 0.001 |
| **Sex (male vs female)** | 1.017 (0.83, 1.247) | 0.869 |
| **BMI, kg/m^2^** | 1.038 (1.01, 1.066) | 0.00618 |
| **ASA classification** |  |  |
| Class Ⅰ | reference |  |
| Class Ⅱ | 3.831 (2.198, 7.467) | < 0.001 |
| Class Ⅲ | 16.969 (9.545, 33.509) | < 0.001 |
| Class Ⅳ | 56.915 (29.894, 117.536) | < 0.001 |
| **Hypertension** | 4.401 (3.59, 5.398) | < 0.001 |
| **Diabetes mellitus** | 2.885 (2.295, 3.601) | < 0.001 |
| **Previous stroke** | 18.863 (15.035, 23.519) | < 0.001 |
| **Coronary heart disease** | 3.651 (2.636, 4.935) | < 0.001 |
| **Myocardial infarction** | 4.109 (1.62, 8.437) | < 0.001 |
| **Heart failure** | 12.102 (3.706, 28.848) | < 0.001 |
| **Atrial fibrillation** | 8.166 (4.19, 14.23) | < 0.001 |
| **Valvular heart disease** | 4.694 (1.85, 9.644) | < 0.001 |
| **Angina pectoris** | 5.414 (2.133, 11.133) | < 0.001 |
| **Peripheral vascular disease** | 7.768 (6.069, 9.843) | < 0.001 |
| **Renal insufficiency** | 1.679 (0.663, 3.437) | 0.209 |
| **Malignant tumor** | 0.689 (0.556, 0.849) | < 0.001 |
| **Preoperative hemoglobin, g/L** | 0.995 (0.99, 1.001) | 0.0873 |
| **Preoperative serum albumin, g/L** | 0.916 (0.897, 0.937) | < 0.001 |
| **Preoperative total bilirubin, μmol/L** | 0.998 (0.993, 1.002) | 0.369 |
| **Preoperative FPG > 6.1mmol/L** | 3.971 (3.22, 4.884) | < 0.001 |
| **NLR > 2.18** | 2.516 (2.05, 3.094) | < 0.001 |
| **PLR > 122** | 0.932 (0.898, 0.965) | < 0.001 |
| **FAR > 0.075** | 2.342 (1.892, 2.913) | < 0.001 |
| **Preoperative thrombin time, s** | 0.961 (0.883, 1.028) | 0.341 |
| **Preoperative ACEI drugs** | 3.835 (2.528, 5.569) | < 0.001 |
| **Preoperative ARB drugs** | 3.040 (2.163, 4.156) | < 0.001 |
| **Preoperative β blockers** | 4.125 (3.041, 5.483) | < 0.001 |
| **Preoperative calcium channel blockers** | 4.617 (3.737, 5.686) | < 0.001 |
| **Preoperative steroids** | 1.724 (1.241, 2.336) | < 0.001 |
| **Perioperative non-steroidal drugs** | 1.698 (1.352, 2.151) | < 0.001 |
| **Preoperative MAP, mmHg** | 1.047 (1.039, 1.056) | < 0.001 |
| **Emergent surgery** | 6.703 (5.031, 8.780) | < 0.001 |
| **Surgery type** |  |  |
| ENT | reference |  |
| Obstetrics and gynecology | 0.575 (0.273, 1.129) | 0.122 |
| Abdominal surgery |  |  |
| Orthopedics | 1.559 (1.02, 2.459) | 0.046 |
| Stomatology | 1.037 (0.506, 2.002) | 0.917 |
| Urology | 0.744 (0.398, 1.353) | 0.339 |
| General surgery | 0 (0, 0) | 0.93 |
| Other surgeries | 0.347 (0.056, 1.159) | 0.149 |
| Neurosurgery | 5.833 (3.933, 8.99) | < 0.001 |
| Thoracic surgery | 0.633 (0.309, 1.221) | 0.187 |
| Vascular surgery | 3.304 (1.464, 6.774) | 0.002 |
| **ln (Surgery length)** | 2.478 (2.045, 3.000) | < 0.001 |
| **Intraoperative Steroids** | 1.257 (0.959, 1.676) | 0.108 |
| **Blood products usage** | 1.975 (1.526, 2.525) | < 0.001 |
| **Crystals (ml/kg/h)** | 0.891 (0.864, 0.917) | < 0.001 |
| **Colloids (ml/kg/h)** | 1.015 (0.975, 1.056) | 0.461 |
| **Morphine equivalents, mg** | 1.003 (1.001, 1.005) | 0.00149 |

Age and surgery length were ln transformed. FPG, NLR, PLR and FAR were transformed to binary data according to the cut-off values. *P*-values were determined using the Wald test. ACEIs, angiotensin-converting enzyme inhibitors; ARBs, angiotensin II receptor blockers; ASA, American Society of Anesthesiologists; BMI, body mass index; CI, confidence interval; ENT, ear, nose and throat; FAR, fibrinogen to albumin ratio; FPG, fasting plasma glucose; MAP, mean arterial pressure; NLR, neutrophil-lymphocyte ratio; OR, odds ratio; PLR, platelet-to-lymphocyte ratio.
