# Supplementary material for: Risk factor analysis and creation of an externally-validated prediction model for perioperative stroke following non-cardiac surgery: A multi-center retrospective and modeling study
Source: PLoS Med. 2025 Mar 21;22(3):e1004539. doi: 10.1371/journal.pmed.1004539 (PMC11927879; doi:10.1371/journal.pmed.1004539)
Supplement: S5 Table — (DOC) [file pmed.1004539.s009.doc]

**Supplementary Table 5 Details for the unadjusted analyses**

| **Variables** | β Coeffcient | OR (95% CI) *P* value | |
| --- | --- | --- | --- |
| **ln Age** | 1.896 | 6.662 (3.844, 11.812) | < 0.001 |
| **ASA classification** |  |  |  |
| Class Ⅰ |  | reference |  |
| Class Ⅱ | 0.391 | 1.478 (0.832, 2.920) | 0.218 |
| Class Ⅲ | 0.692 | 1.998 (1.070, 4.088) | 0.041 |
| Class Ⅳ | 0.958 | 2.607 (1.174, 6.105) | 0.022 |
| **Hypertension** | 0.354 | 1.425 (1.090, 1.861) | 0.009 |
| **Previous stroke** | 1.723 | 5.604 (4.313, 7.242) | < 0.001 |
| **Valvular heart disease,** | 0.93 | 2.535 (0.935, 5.698) | 0.041 |
| **Peripheral vascular disease** | 0.657 | 1.929 (1.421, 2.592) | < 0.001 |
| **Preoperative FPG > 6.1mmol/L** | 0.385 | 1.469 (1.125, 1.912) | 0.004 |
| **FAR > 0.075** | 0.245 | 1.278 (1.003, 1.635) | 0.049 |
| **Preoperative MAP (mmHg)** | 0.018 | 1.018 (1.009, 1.027) | < 0.001 |
| **Surgery type** |  |  |  |
| ENT |  | reference |  |
| Obstetrics and gynecology | -0.613 | 0.542 (0.253, 1.086) | 0.096 |
| Abdominal surgery | -1.306 | 0.271 (0.165, 0.453) | < 0.001 |
| Orthopedics | -0.425 | 0.653 (0.409, 1.071) | 0.082 |
| Stomatology | -0.482 | 0.617 (0.295, 1.220) | 0.179 |
| Urology | -1.138 | 0.32 (0.168, 0.595) | < 0.001 |
| General surgery | -14.907 | 0 (0, 0) | 0.953 |
| Other | -1.463 | 0.231 (0.037, 0.782) | 0.047 |
| Neurosurgery | 0.636 | 1.890 (1.217, 3.024) | 0.006 |
| Thoracic surgery | -1.255 | 0.285 (0.136, 0.568) | 0.001 |
| Vascular surgery | -1.497 | 0.224 (0.093, 0.497) | < 0.001 |
| **Emergent surgery** | 1.049 | 2.853 (1.929, 4.169) | < 0.001 |
| **ln (Surgery length)** | 0.619 | 1.857 (1.396, 2.471) | < 0.001 |

Age and surgery length were ln transformed. FAR was transformed to binary data according to the cut-off value. *P*-values were determined using the Wald test. ASA, American Society of Anesthesiologists; CI, confidence interval; ENT, ear, nose and throat; FPG, fasting plasma glucose; MAP, mean arterial pressure; OR, odds ratio; FAR, fibrinogen to albumin ratio.
