# Supplementary material for: Risk factor analysis and creation of an externally-validated prediction model for perioperative stroke following non-cardiac surgery: A multi-center retrospective and modeling study
Source: PLoS Med. 2025 Mar 21;22(3):e1004539. doi: 10.1371/journal.pmed.1004539 (PMC11927879; doi:10.1371/journal.pmed.1004539)
Supplement: S6 Table — (DOC) [file pmed.1004539.s010.doc]

**Supplementary Table 6 Details of rejected variables**

| **Variables** | OR (95% CI) | *P* value |
| --- | --- | --- |
| **BMI, kg/m^2^** | 0.994 (0.962, 1.026) | 0.699 |
| **Diabetes mellitus** | 1.238 (0.945, 1.611) | 0.117 |
| **Coronary heart disease** | 1.188 (0.816, 1.691) | 0.354 |
| **Myocardial infarction** | 1.266 (0.481, 2.753) | 0.589 |
| **Heart failure** | 1.929 (0.534, 5.355) | 0.254 |
| **Atrial fibrillation** | 1.549 (0.729, 2.984) | 0.220 |
| **Angina pectoris** | 1.490 (0.547, 3.374) | 0.383 |
| **Malignant tumor** | 0.973 (0.731, 1.292) | 0.849 |
| **Preoperative serum albumin, g/L** | 0.981 (0.955, 1.008) | 0.164 |
| **NLR > 2.18** | 1.145 (0.887, 1.476) | 0.299 |
| **PLR > 122** | 1.263 (0.992, 1.611) | 0.059 |
| **Preoperative ACEI drugs** | 1.124 (0.713, 1.706) | 0.597 |
| **Preoperative ARB drugs** | 0.991 (0.675, 1.421) | 0.962 |
| **Preoperative β blockers** | 1.345 (0.943, 1.887) | 0.093 |
| **Preoperative calcium channel blockers** | 1.221 (0.939, 1.585) | 0.135 |
| **Preoperative steroids** | 1.404 (0.978, 1.970) | 0.057 |
| **Blood products usage** | 1.054 (0.777, 1.417) | 0.732 |
| **Crystals, ml/kg/h** | 1.008 (0.972, 1.044) | 0.669 |
| **Morphine equivalents, mg** | 1.000 (0.998, 1.003) | 0.826 |

NLR and PLR were transformed to binary data according to the cut-off value. *P*-values were determined using the Wald test. ACEIs, angiotensin-converting enzyme inhibitors; ARBs, angiotensin II receptor blockers; BMI, body mass index; CI, confidence interval; NLR, neutrophil-lymphocyte ratio; OR, odds ratio; PLR, platelet-to-lymphocyte ratio.
