# Supplementary material for: Risk factor analysis and creation of an externally-validated prediction model for perioperative stroke following non-cardiac surgery: A multi-center retrospective and modeling study
Source: PLoS Med. 2025 Mar 21;22(3):e1004539. doi: 10.1371/journal.pmed.1004539 (PMC11927879; doi:10.1371/journal.pmed.1004539)
Supplement: S7 Table — (DOC) [file pmed.1004539.s011.doc]

**Supplementary Table 7 Scales for the 13 predictor variables**

| Variables | Value | Point |
| --- | --- | --- |
| **ln Age** | 2.8 | 0 |
|  | 3.0 | 4 |
|  | 3.2 | 8 |
|  | 3.4 | 12 |
|  | 3.6 | 16 |
|  | 3.8 | 20 |
|  | 4.0 | 24 |
|  | 4.2 | 28 |
|  | 4.4 | 32 |
|  | 4.6 | 36 |
| **ASA classification** | Class Ⅰ | 0 |
|  | Class Ⅱ | 4 |
|  | Class Ⅲ | 8 |
|  | Class Ⅳ | 10 |
| **Hypertension** | Yes | 5 |
|  | No | 0 |
| **Previous stroke** | Yes | 18 |
|  | No | 0 |
| **Valvular heart disease** | Yes | 10 |
|  | No | 0 |
| **Preoperative FPG > 6.1 mmol/L** | Yes | 5 |
|  | No | 0 |
| **Preoperative FAR > 0.075** | Yes | 4 |
|  | No | 0 |
| **Preoperative steroid hormones** | Yes | 4 |
|  | No | 0 |
| **Preoperative β blockers** | Yes | 5 |
|  | No | 0 |
| **Preoperative MAP, mmHg** | 20 | 0 |
|  | 40 | 3 |
|  | 60 | 6 |
|  | 80 | 9 |
|  | 100 | 13 |
|  | 120 | 16 |
|  | 140 | 19 |
|  | 160 | 22 |
|  | 180 | 25 |
|  | 200 | 28 |
| **Surgery type** | ENT | 92 |
|  | Obstetrics and gynecology | 87 |
|  | Abdominal surgery | 80 |
|  | Orthopedics | 89 |
|  | Stomatology | 88 |
|  | Urology | 81 |
|  | General surgery | 0 |
|  | Other | 78 |
|  | Neurosurgery | 100 |
|  | Thoracic surgery | 80 |
|  | Vascular surgery | 84 |
| **Emergent surgery** | Yes | 10 |
|  | No | 0 |
| **ln (Surgery length)** | 4.0 | 0 |
|  | 4.5 | 3 |
|  | 5.0 | 6 |
|  | 5.5 | 9 |
|  | 6.0 | 12 |
|  | 6.5 | 15 |
|  | 7.0 | 18 |
|  | 7.5 | 21 |

Age and surgery length were ln transformed. ASA, American Society of Anesthesiologists; ENT, ear, nose and throat; FAR, fibrinogen to albumin ratio; FPG, fasting plasma glucose; MAP, mean arterial pressure.
