# Supplementary material for: Risk factor analysis and creation of an externally-validated prediction model for perioperative stroke following non-cardiac surgery: A multi-center retrospective and modeling study
Source: PLoS Med. 2025 Mar 21;22(3):e1004539. doi: 10.1371/journal.pmed.1004539 (PMC11927879; doi:10.1371/journal.pmed.1004539)
Supplement: Attachment — Submitted filename: Response to reviewers.docx [file pmed.1004539.s012.docx]

ACADEMIC EDITOR COMMENTS

Many of the comments are important but addressable. I think the following are critical: 

1) Comment #1 (‘perioperative aspirin’) by Reviewer 4 - very important and needs a clear response

2) Comment #9, #10 and #11 by Reviewer 2

Response: Many thanks for your hard work handling our manuscript. We have supplied clear responses to all the reviewers’ comments including the critical comments as you mentioned above.

EDITORIAL COMMENTS

In line with the reviewers' comments, we strongly feel that validation in a non-Chinese cohort would be ideal, but are not going to make this a mandatory requirement. Please consider whether including the final model variables in the abstract would be beneficial without overloading the structure of the abstract.

Response: It is very difficult to make the validation in a non-Chinese cohort for us by now, but this is a very inspiring idea, and we will make efforts to complete this in our next research. We have added the final model variables in the revised abstract in page 1 in line13-19 according to your suggestion, and highlighted them in yellow.

FINCANCIAL DISCLOSURE

The funding statement should include: specific grant numbers, initials of authors who received each award, URLs to sponsors’ websites. Also, please state whether any sponsors or funders (other than the named authors) played any role in study design, data collection and analysis, the decision to publish, or preparation of the manuscript. If they had no role in the research, include this sentence: “The funders had no role in study design, data collection and analysis, decision to publish, or preparation of the manuscript.”

Response: We have added the initials of authors who received each award in the funding statement. And on other sponsors or funders provide financial support to this research. We have added the sentence “The funders had no role in study design, data collection and analysis, decision to publish, or preparation of the manuscript.” in page 21 in line 429-433.

COMEPTING INTEREST

All authors must declare their relevant competing interests per the PLOS policy, which can be seen here: https://journals.plos.org/plosmedicine/s/competing-interests
For authors with ties to industry, please indicate whether any of the interests has a financial stake in the results of the current study.

Response: All the authors have seen the policy of competing interest above and we have made a declaration of “Conflict of interests” in the manuscript.

GENERAL COMMENTS
1) Please include page numbers and line numbers in the manuscript file. Use continuous line numbers (do not restart the numbering on each page). For review purposes, we started counting the Abstract as page 1.

Response: We have added the line number and page number starting with the Abstract.

2) Please cite the reference numbers in square brackets (e.g., “We used the techniques developed by our colleagues [19] to analyze the data”). Citations should be preceding punctuation.

Response: We have revised the reference based on this comment.

3) Please cite your Supporting Information as outlined here: <https://journals.plos.org/plosmedicine/s/supporting-information>

Response: We have cited our supplementary files as Supporting Information in the main text. If you still have any problem, please do not hesitate to contact us. We will try our best to solve them.

4) Please ensure that the study is reported according to the TRIPOD guideline and include the completed TRIPOD checklist as Supporting Information. When completing the checklist, please use section and paragraph numbers, rather than page numbers. Please add the following statement, or similar, to the Methods: "This study is reported as per the Transparent reporting of a multivariable prediction model for individual prognosis or diagnosis (TRIPOD): The TRIPOD statement (S1 Checklist).”

Response: We thank the editor for pointing out this issue, and we have completed the TRIPOD checklist, supplied it as Supplementary Table 1 in the revision, and relevant statements were added in the Methods section in line 126-128.

ABSTRACT

1) PLOS Medicine requests that main results are quantified with 95% CIs as well as p values. When reporting p values please report as p<0.001 and where higher as the exact p value p=0.002, for example. For the purposes of transparent data reporting, if not including the aforementioned please clearly state the reasons why not.

Response: We thank the editor for pointing out this issue. We have reported the p values for results quantified with 95% CIs, and all the p values are reported as requested in page 1 in line 19-20.

2) Throughout, suggest reporting statistical information as follows to improve clarity for the reader “22% (95% CI [13%,28%]; p</=)”. Please amend throughout the abstract and main manuscript. Please note the use of commas to separate upper and lower bounds, as opposed to hyphens as these can be confused with reporting of negative values.

Response: We thank the editor for pointing out this issue. We have amended the statistical information throughout the abstract and main manuscript. We retained the use of hyphens as there are no negative values reported in the manuscript.

3) When a p value is given, please specify the statistical test used to determine it. 

Response: We thank the editor for pointing out this issue, and we have specified the statistical test used to determine a given p value in the manuscript.

4) Please structure your abstract using the PLOS Medicine headings (Background, Methods and Findings, Conclusions). Please combine the Methods and Findings sections into one section, “Methods and findings”.

Response: We thank the editor for pointing out this issue, and based on this comment, we have revised the abstract.

5) Please ensure that all numbers presented in the abstract are present and identical to numbers presented in the main manuscript text.

Response: We thank the editor for pointing out this issue, and we have vetted the manuscript to ensure that all numbers presented in the abstract are identical to numbers presented in the main manuscript text.

6) Please include the age of the study population.

Response: We thank the editor for pointing out this issue, and we have included the age of the study population in the results section in the main text.

7) Please include the important dependent variables that are adjusted for in the analyses.

Response: We thank the editor for pointing out this issue, and we have included the important dependent variables that are adjusted for in the Model development and validation section in page 1 in line 13-19.

8) In the last sentence of the Abstract Methods and Findings section, please describe the main limitation(s) of the study's methodology.

Response: We have described the main limitations of the study's methodology in the last sentence of the revised Abstract Methods and Findings section in page 2 in line 27-30.

9) Abstract Conclusions:
* Please interpret the study based on the results presented in the abstract, emphasizing what is new without overstating your conclusions.
* Please avoid assertions of primacy ("novel...."). Please temper claims of primacy of results by stating, "to our knowledge" or something similar.

Response: We have deleted the assertion of primacy ("novel....") in the abstract conclusion.

10) Please ensure that the abstract is reported according to the TRIPOD guideline.

Response: We ensure that the abstract is reported according to the TRIPOD guideline.

AUTHOR SUMMARY

At this stage, we ask that you include a short, non-technical Author Summary of your research to make findings accessible to a wide audience that includes both scientists and non-scientists. The Author Summary should immediately follow the Abstract in your revised manuscript. This text is subject to editorial change and should be distinct from the scientific abstract. Please see our author guidelines for more information: https://journals.plos.org/plosmedicine/s/revising-your-manuscript#loc-author-summary.

The summary should include 2-3 single sentence, individual bullet points under each of the questions. The last bullet under ‘What Do These Findings Mean?’ point should describe the main limitation of the study's methodology.

It may be helpful to review currently published articles for examples which can be found on our website here https://journals.plos.org/plosmedicine/

Response: We have included a short, non-technical Author Summary in the revision in page 2-4.

INTRODUCTION

If there has been a systematic review of the evidence related to your study (or you have conducted one), please refer to and reference that review and indicate whether it supports the need for your study.

Response: We have added a systematic review related to this study in the revised Introduction in page 6 in line 106-109, which is published in The Lancet Neurology (Oct, 2023) by Professor Philip James Devereaux, the academician of Canadian Academy of Health Sciences.

METHODS AND RESULTS

1) PLOS Medicine requests that main results are quantified with 95% CIs as well as p values. When reporting p values please report as p<0.001 and where higher as the exact p value p=0.002, for example. For the purposes of transparent data reporting, if not including the aforementioned please clearly state the reasons why not. Please include any important dependent variables that are adjusted for in the analyses. We suggest reporting statistical information as detailed above – see under ABSTRACT.

Response: We thank the editor for pointing out this issue. We have amended the statistical information throughout the manuscript as requested.

2) Please present numerators and denominators for percentages, at least in the Tables [not necessarily each time they're mentioned].

Response: We thank the editor for pointing out this issue. The numerators for percentages were presented in each cell and the denominators for percentages were presentd in the first row of the tables. We also have reported numerators and denominators for percentages in the main manuscript.

3) The link for the 301 Perioperative Stroke Risk Calculator (301PSRC) cannot be accessed. Please check.

Response: We are very sorry that we have changed the website of the 301PSRC online calculator to <http://139.196.204.68:8087/>.

3) Please report the Methods and Results according to the TRIPOD Checklist: Prediction Model Development and Validation.

Response: We thank the editor for pointing out this issue, and we have reported the Methods and Results according to the TRIPOD Checklist.

DISCUSSION

Please ensure to present and organize the Discussion as follows: a short, clear summary of the article's findings; what the study adds to existing research and where and why the results may differ from previous research; strengths and limitations of the study; implications and next steps for research, clinical practice, and/or public policy; one-paragraph conclusion. Please avoid the use of subheadings such that the discussion reads as continuous prose.

Response: We ensure that we have presented and organized the Discussion as you stated above.

1) Please temper claims of primacy of results by stating, "to our knowledge" or something similar.
(e.g. "We also discovered several new preoperative risk factors...."). 

Response: We have tempered claims of primacy of results in the revision.

2) Please temper claims of “preeminent”, “excellent” and the like.

Response: We have tempered the claims of preeminent”, “excellent” and the like in the revision.

TABLES

1) Throughout please ensure that each table is affiliated to an appropriate title and caption which clearly describes the table content without the need to refer to the text.

Response: We thank the editor for pointing out this issue, and we have modified the caption of Table 1.

2) Please ensure that all abbreviations including those used for statistical reporting are clearly defined in the caption or footnote.

Response: We thank the editor for pointing out this issue, and we have defined all abbreviations including those used for statistical reporting in the caption.

3) Please ensure that where confidence intervals are reported, p values are also reported as described above. Please note the use of commas to separate upper and lower bounds, as opposed to hyphens as these can be confused with reporting of negative values.

Response: We thank the editor for pointing out this issue. We have amended the statistical information throughout the manuscript. We retained the use of hyphens as there are no negative values reported in the manuscript.

FIGURES

1) For all Figures, please ensure that you have complied with our figures requirements http://journals.plos.org/plosmedicine/s/figures.

Response: We thank the editor for pointing out this issue. For all the Figures, we have complied with the figures requirements.

2) Please consider avoiding the use of red and green in order to make your figure more accessible to those with colour blindness. 

Response: We thank the editor for pointing out this issue, and we have modified Figure 3 to make it more accessible to those with colour blindness.

3) Please in the figure legend/description, define abbreviations used in each figure (including those in Supporting Information files).

Response: We thank the editor for pointing out this issue, and we have defined abbreviations used in each figure in page 26.

4) Please provide titles, legends and descriptions for all figures (including those in Supporting Information files).

Response: We thank the editor for pointing out this issue. We have provided titles, legends and descriptions for all figures in the manuscript in page 26, including those in Supporting Information files.

5) Figure 1: The graphs are rather small and the details are not easy to read. Please revise.

Response: We thank the editor for pointing out this issue. We have modified Figure 1 and Supplementary Figure 1 to make the figures easier to read.

6) Figure 1: In Figure 1 A, please explain the different scales for the different factors. What does 'In age' mean? Please remove 'Lorem Ipsum' from Graph D. In Graph D, the different colors, especially the two gray colors, are difficult to distinguish - please revise.

Response: We thank the editor for pointing out this issue. We have explained the different scales for the different factors in Supplementary Table 6. ‘ln age’ means the ln transformation of age. We have removed 'Lorem Ipsum' from Graph D, and have modified Graph D as well.

7) Figure 3: Please provide references for the models being compared.

Response: We thank the editor for pointing out this issue, and we have provided references for the models being compared in the caption of Figure 3.

SUPPLEMENTARY MATERIAL

1) For supplementary figures and tables, please see the general comments under TABLES and FIGURES (color, abbreviations, titles, descriptions, etc.) and amend accordingly.

Response: We thank the editor for pointing out this issue, and we have amended related figures and tables accordingly.

2) We suggest reporting statistical information as detailed above – see under ABSTRACT. Please define all numerical values.

Response: We thank the editor for pointing out this issue, and we have reported statistical information as suggested.

3) As for the main manuscript, please indicate whether analyses are adjusted to help facilitate transparent data reporting please also detail the factors adjusted for and present the unadjusted analyses for comparison. If not, please clearly state the reasons why not.

Response: We thank the editor for pointing out this issue. We have indicated the factors adjusted for in the manuscript, and have also presented the detailed unadjusted analyses for comparison in Supplementary Table 5.

REFERENCES

1) PLOS uses the numbered citation (citation-sequence) method and first six authors, et al.

2) Please ensure that journal name abbreviations match those found in the National Center for Biotechnology Information (NCBI) databases (http://www.ncbi.nlm.nih.gov/nlmcatalog/journals), and are appropriately formatted and capitalised.

3) Where website addresses are cited, please specify the date of access. 

4) Please also see https://journals.plos.org/plosmedicine/s/submission-guidelines#loc-references for further details on reference formatting.

Response: We thank the editor for pointing out this issue. We have revised REFERENCES based on this comment.

Comments from the reviewers:

Reviewer #1: The author identified the risk factors and developed prediction model for perioperative stroke following non-cardiac surgery. This study could be expected to have an important impact on daily clinical management for prevention of perioperative stroke. However, there are some questions and comments about contents of the manuscript.

Response: Thanks very much for your positive comments.

In the Introduction section, there is no need to explain what "ischemic stroke" means.

Response: We have deleted the explanation of "ischemic stroke" in the revision.

The description of the 301 PSRC was in the last paragraph in the result section, but the analysis using this software was described in the preceding 6th paragraph and figure 3. The author should reconsider the order of the contents about the 301 PSRC. 

Response: We have adjusted the order of the contents about the 301 PSRC in the revision.

As the authors mentioned, it would be useful to focus on intraoperative factors to reduce the risk of perioperative stroke and to detect perioperative stroke onset earlier. However, if the reason for the higher predictive ability of this model compared to previous models is due to the employment of intraoperative factors in this study, then it is not surprising that this model has a higher predictive ability. For the purpose of preoperative stroke risk prediction only, a model consisting only of information available before the start of surgery is favorable. The authors should mention these points as limitations of this study.

Response: This question is very profound and triggers our deep thinking. In fact, perioperative stroke nearly occurs after surgery, especially on postoperative 1-3 days. Intraoperative factors such as surgery duration, bleeding, intraoperative medication and so on may lead to the occurrence of perioperative stroke. Therefore, in analyzing risk factors and constructing predictive models, we included the intraoperative variables, which enabled our model to not only predict perioperative stroke before surgery but also could make more accurate prediction after surgery. Thus, we believe this is one of our advantages compared with previous researches but not a limitation.

Reviewer #2: "Risk Factor Analysis and Creation of an Externally-Validated Prediction Model for Perioperative Stroke Following Non-Cardiac Surgery" describes the development and (external) validation of a risk (nomogram) model, for the prediction of perioperative stroke risk. The model was developed on a cohort of some 223,000 non-cardiac surgical patients from 2008-2019 attending the First Medical Center of Chinese PLA General Hospital, and externally validation on data from two other Chinese hospitals. An AUC of about 0.90 was reported on all three cohorts.

While the scale of the study is impressive, some issues might be considered:

1. In the Abstract, AUC values were given to various precisions (i.e. 0.91, 0.9, 0.894). The number of decimal places/significant figures might be standardized here and throughout the manuscript if appropriate. The 95% CIs for the external validation cohort AUCs might also be stated.

Response: We thank the reviewer for pointing out this issue, and we have standardized AUC values in the manuscript. We have also reported 95% CIs for the external validation cohort AUCs in the manuscript.

2. In the Study design and cohorts section, various exclusion criteria were described. A flowchart showing the number of patients eliminated according to each of these criteria might be included as a figure if possible.

   Response: We thank the reviewer for pointing out this issue, and we have included the number of patients eliminated according to each exclusion criteria in the study flow chart in Supplementary Figure 1.

3. In the Study design and cohorts section, "missing data for any variables" was stated as an exclusion criteria. However, imputation for variables with <20% missing data is later mentioned, which implies that patients with missing data for some variables were included. This might be clarified.

Response: We are sorry that we reported the exclusion criteria erroneously, we have corrected the exclusion criteria in the method section in page 7, as well as in Supplementary Figure 1.

4. In the Predictor variables section, it is stated that multiple imputation was used for variables with <20% missing data. Details of this multiple imputation, including the method (e.g. MCMC, single value, etc.) and any parameters might be briefly mentioned.

Response: We thank the reviewer for pointing out this issue, and we have specified the method used for multiple imputations in the Predictor variables section in page 8.

5. In the Model development and validation section, it is stated that stepwise elimination was used to select the final 14 variables. The p-values of rejected variables might be included in a table, possibly in supplementary material.

Response: We thank the reviewer for pointing out this issue, and we have included the CIs and p-values of the rejected variables in Supplementary Table 5.

6. In the Statistical analyses section, external data was stated to be collected from Nanfang and Henan. It might be stated whether these external cohorts followed the same exclusion criteria as the development cohort.

Response: We thank the reviewer for pointing out this issue, and we have stated in the Method section that the external cohorts followed the same exclusion criteria as the development cohort.

7. The missing data percentage for each variable might be included in Table 2, if possible.

Response: We thank the reviewer for pointing out this issue, and we have included the missing data percentage for each variable in Table 2.

8. In the Prediction Model for Perioperative Stroke section, a nomogram (Figure 1A) was introduced, and a cutoff of 158.3 points stated. The construction of this nomogram (and cutoff, including specific definition of "high-risk" - e.g. percentage chance of developing stroke within 30 days) might be briefly described/referenced, possibly in supplementary material.

Response: We thank the reviewer for pointing out this issue, and we have explained the construction of this nomogram including cutoff, definition of "high-risk", and the percentage chance of developing stroke.

9. In the Comparison with existing stroke models section, it is stated that the developed model exhibited improved discrimination (AUC=0.91) compared to a number of existing stroke models. However, this appears to be analyzed only on the developmental cohort, which might present the developed model with an advantage (since it was developed on that cohort itself). As such, it would be strongly recommended to also compare the developed model against existing stroke models, on the external validation cohorts.

Response: We have compared the prediction model against the ATRIA stroke risk score and the model proposed by Lip and colleagues using the data of Nanfang Hospital and Henan Provincial People's Hospital, as the prediction variables current smoker, chronic obstructive pulmonary disease, hematocrit, and preoperative serum sodium in the other 3 models were absent in the two external cohorts.
10. Moreover, it is not clear that the four models compared against (Wolf et al., ATRIA, Lip et al., Mashour et al.) are the most appropriate/relevant, partly as they are fairly dated (with ATRIA being the newest, from 2013). It might thus also be strongly recommended to include newer models (e.g. "Cardiovascular Risk Scores to Predict Perioperative Stroke in Noncardiac Surgery", Wilcox et al., Stroke 2019; "Development and Validation of a Prediction Model for Stroke, Cardiac, and Mortality Risk After Non‐Cardiac Surgery", Woo et al., JAHA 2021), and also the most commonly-used models such as CHA2DSVASc, ACS-NSQIP, MICA etc., especially since some of their AUCs are given in the Discussion section, but are not analyzed in Figure 3 and the relevant results section) in the comparison.

Response: We thank the reviewer for pointing out this issue. Wilcox et al used Cardiovascular Risk Scores to predict perioperative stroke, of which the factor “dependent functional status” was a crucial variable. Unfortunately, the variable “dependent functional status” is absent in the three hospitals in current study, and we are not able to compare the current model against model by Wilcox et al. But the variables used by Woo et al were all available in the First Medical Center of Chinese PLA General Hospital, thus we have added the model by Woo et al in the comparison in page 15. We have stated the reason of not comparising the CHA2DSVASc, ACS-NSQIP and MICA were stated in the Discussion section in page 19.

11. Additionally, the external validation data is also derived exclusively from Chinese populations (Nanfang/Henan). If possible, it should be considered to also externally validate on non-Chinese/international datasets, to determine whether the performance improvement is specific to ethnicity.

Response: We thank the reviewer for this inspiring suggestion. We have also considered this issue, but due to a lack of communication and cooperation before, this idea has not been truly implemented. We will try our best to conduct externally validate on non-Chinese/international datasets in our next research. We have added this deficiency as a limitation of our study in the revised Discussion.

12. In the Practical Application of Risk Model section, a link to the 301PSRC online calculator was given as http://211.166.249.201:6003. This link however does not appear to display the risk calculator. This might be addressed.

Response: We are very sorry that we have changed the website of the 301PSRC online calculator to <http://139.196.204.68:8087/>.

13. In the Discussion section, it is stated that "We incorporated four existing models into our database for perioperative stroke risk prediction, without the ACS-NSQIP Surgical Risk Calculator and MICA Risk Score as the discrepancy of the indicators". This sentence might be clarified.

Response: We had explained this sentence in the revised Discussion in page 19 in line 380-381. The ACS-NSQIP Surgical Risk Calculator did not predict stroke as an independent outcome and could not be included in our database for prediction. And the MICA Risk Score is designed mainly to predict perioperative myocardial infarction (MI) and cardiac arrest (CA), but not to predict perioperative stroke.

14. In Table 2, the actual variables might be bolded for clarity, with the categories within variables (e.g. for Surgery Type) left unbolded and possibly offset.

Response: We thank the reviewer for pointing out this issue, and we have modified Table 2 as suggested.

15. In Table 2, "Emergent surgery" might be "Emergency surgery".

Response: We thank the reviewer for pointing out this issue, and we have revised "Emergent surgery" to "Emergency surgery" in table 1 and table 2.

16. The chart title for Figure 1D (Decision Curve Analysis) is "Lorem Ipsum" placeholder text. This might be replaced with the actual intended title.

Response: We are very sorry for this mistake and we have removed "Lorem Ipsum" in the revised Figure 1D.

Reviewer #3: The present research is clinically relevant, thank you for letting me peer review it. The following two points are my comments.
1.  I think it is important for authors to have developed an application that is easy to use, and I think it is expected that this risk model will be used before non-cardiac surgery especially in China through the newly developed application. However, I could not identify the application from the link presented in the manuscript. Could you correct this so that I can see what that application is?

Response: We are very sorry that we have changed the website of the 301PSRC online calculator to <http://139.196.204.68:8087/>.

2.  The exclusion criteria include patients with missing data for any variables, but the Predictor Variables subsection states that: 
"Variables with ≥20% missing data were excluded. We used multiple imputations to impute missing values for variables with <20% missing data", 
which is inconsistent.
I could not understand whether this modeling study was based on complete data analysis or imputed data analysis.

Response: We are sorry that we reported the exclusion criteria erroneously. The modeling study was based on imputed data analysis, and we have corrected the exclusion criteria in the method section in page 7, as well as in Figure 1.

Reviewer #4: Yulong Ma and colleagues reported methods and results of the development of a prediction model for the risk of perioperative stroke within 30 days after noncardiac surgery, analyzing administrative hospital data from three Chinese hospitals. Data from adult patients that underwent noncardiac surgery from 2008-2019 at The First Medical Center of Chinese PLA General Hospital were used as derivation cohort; the model was then externally validated using the hospital records of two other Chinese centres. They also compared the predictive performance of their model with that of other existing prediction models. They also translated their model into a risk calculator for clinical use.

The study itself could add to our understanding of risk factors for perioperative stroke after noncardiac surgery and could eventually offer a prediction tool that, after further validation (prospectively and externally in other countries), could even enter clinical practice. The statistical methods the investigators used appear quite rigorous. There is also to acknowledge that, among existing cohort studies with multivariable analyses of perioperative stroke predictors, the databases here analyzed would provide the most recent data (see Marcucci et al., Lancet Neurol 2023; 22: 946-58). There are however major concerns with the study; in particular one possible major flow that I recommend to address before considering the article for publication.

Response: Thanks very much for your positive comments. We have carefully revised or responded to your concerns as follows.

Major concern
1. Among the variables they consider as possible risk factors, the authors include what they name "perioperative aspirin". The only definition they provide for this is that "the patient received aspirin medication perioperatively." What "perioperatively" mean? If this means (as usually) that they considered when patients took aspirin any time around surgery, inclduing pre-, potentially intra-, and post-operatively (for example on any day after surgery they spent in hospital, or even only on the first few days after surgery), this means this could be aspirin given BECAUSE the patient had an intra- or postoperative stroke. This means, that to include perioperative aspirin as a risk factor or predictor for stroke is reverse association and therefore biased analysis. If this is what they did, the very strong association between perioperative aspirin and stroke shown in the univariable and multivariable models is then easily explained; as explained is the outstanding
discrimination performance (AUC>0.9) the authors found for their model (exceptional for a prognostic model). If this is what they did, the authors should remove this variable from the studied risk factors, and re-run the multivariable analyses, and provide a new model and risk calculator.
If, instead, "perioperative aspirin" had a different meaning (for example, it is preoperative aspirin) this should be amended or clarified. 

Response: We thank the reviewer for this crucial question, which we had not elaborated clearly in the Methods. We apologize for the confusion caused by our unclear description. “The patient received aspirin medication perioperatively” means that the patients took aspirin both pre-operatively and post-operatively for anti-coagulation medication, not because the patient had an intra- or postoperative stroke. We have clarified this variable in the revised Methods in page 8 in line 161-162.

Other major points
2. The rationale for selecting the models they selected for comparison, and not other existing ones, should be better explained. Only one of the selected models was developed to predict perioperative stroke; the other ones were models for non-operative stroke; two of them for the risk of stroke in patients with atrial fibrillation. For instance, it is unclear why the authors did not compare their model with that of Woo et al. (reference 10), which is a prediction model developed in a similar way as the one here developed but from an US hospital database (i.e., the comparison would have been more relevant and interesting). In the introduction the authors mention Woo et al. among the existing models which have the limitation of predicting "various perioperative complications of the cerebrovascular system" at once; in fact, Woo et al. does include also a specific prediction model for stroke. If not selected as a comparator model, the authors could at least discuss their
findings compared to those of Woo et al.'s findings.

Response: We thank the reviewer for pointing out this issue. We have added the model by Woo et al in the comparison.

On this point, the sentence they include in the Discussion ("We incorporated four existing models,13-15 into our database for perioperative stroke risk prediction, without the ACS-NSQIP Surgical Risk Calculator11 and MICA Risk Score12 as the discrepancy of the indicators") is not clear, and would require rephrasing or explanation. 

Response: We had explained this sentence in the revised Discussion in page 19 in line 380-381. The ACS-NSQIP Surgical Risk Calculator did not predict stroke as an independent outcome and could not be included in our database for prediction. And the MICA Risk Score is designed mainly to predict perioperative myocardial infarction (MI) and cardiac arrest (CA), but not to predict perioperative stroke.

3. If the model confirms its very good performance even once point 1 is addressed, still, the authors should recommend caution in the use of their model and risk calculator. This remains a model developed retrospectively from administrative hospital databases. Furthermore, the fact that the model was developed and externally validated only in a Chinese population and hospital system might eventually prove irrelevant, but we will not know it until it is externally validated in different countries/hospital systems. The authors did acknowledge the lack of prospective validation among the study limitations. My recommendation would be to mitigate the call for use of their model and online calculator including considerations of these limitations in the Conclusions in the abstract and in the text.

Response: We thank the reviewer for pointing out this issue, which is very important. We have added the deficiency of without external validation in other countries/hospital systems as a limitation of our study in the revised Discussion. And we have mitigated the call for use of our model and online calculator in the Conclusions in the abstract and in the text.

4. The definition of the timing of their outcome should be more accurate. Did the "within 30 days following surgery" include the day of surgery?

Response: We thank the reviewer for pointing out this issue. The "within 30 days following surgery" did not include the day of surgery.

5. To include a Decision Curve Analysis seems a very interesting and possibly helpful approach. The authors should better explain what they did (even including some of the more detailed methods in the online supplementary material) and how the reader should interpret the results.

Response: We thank the reviewer for this suggestion, and we have further explained the results of Decision Curve Analysis in the main manuscript in page 13.

Minor comments
6. Regarding the sentence in the Introduction: "Unfortunately, most risk factors to date entail preoperative indicators due to limited accessibility to intra-operative indicators." I would not necessarily put a lot of emphasis on intraoperative predictors since they might help refining the prediction but at the methodological cost of possibly introducing a bias. Some of the perioperative strokes are occurring during the procedure (while the patient is sedated) and what we include as a predictor (for example intraoperative hemodynamics) could in fact be a consequence of or expression of the stroke that is happening. This is particularly possible when the prediction model is based on administrative databases (and no actual date of the event can be used in the analysis) and not based on prospectively collected data where the time relationship between exposure and outcome can be taken into account.

Response: We thank the reviewer for pointing out this issue, and we have deleted this sentence from the revised Introduction.

7. In the evaluation of prognostic models, what the Hosmer-Lemeshow test assesses is typically called 'calibration' (as opposed to discrimination) and not "model accuracy".

Response: We thank the reviewer for pointing out this issue, and we have corrected the description in the manuscript.

8. Table 1 is very difficult to read. The authors (or editors) should consider to change the formatting. One way would be to have in this Table 1 only the description of the derivation (and internal validation) cohort; and have a similar supplementary table for the characteristics of the other two cohorts.

Response: We thank the reviewer for pointing out this issue, and we have changed the formatting to make Table 1 easier to read.

9. Table 1 is introduced in the text as if it is showing only the patient "demographics", but this is not the case.

Response: We thank the reviewer for pointing out this issue, and we have revised the discription of Table 1 in the manuscript.

General editorial requests


1) Please ensure that the paper adheres to the PLOS Data Availability Policy (see http://journals.plos.org/plosmedicine/s/data-availability), which requires that all data underlying the study's findings be provided in a repository or as Supporting Information. For data residing with a third party, authors are required to provide instructions with contact information for obtaining the data. PLOS journals do not allow statements supported by "data not shown" or "unpublished results." For such statements, authors must provide supporting data or cite public sources that include it.

Response: The data used and analyzed during the current study are stored in a internal server of the First Medical Center of Chinese PLA General Hospital, and data copy is forbidden. We're sincerely looking forward to cooperate with scholars from all over the world, and the data are available within the scope of the First Medical Center of Chinese PLA General Hospital, either from the corresponding author or the Medical Ethics Committee of The First Medical Center of Chinese PLA General Hospital upon reasonable request. If you still have any problems, please do not hesitate to contact us. We will try our best to solve them. Have a nice day. Best regards.

2) In addition, we request that you upload any figures associated with your paper as individual TIF or EPS files with 300dpi resolution at resubmission; please read our figure guidelines for more information on our requirements: http://journals.plos.org/plosmedicine/s/figures. While revising your submission, please upload your figure files to the PACE digital diagnostic tool, https://pacev2.apexcovantage.com/. PACE helps ensure that figures meet PLOS requirements. To use PACE, you must first register as a user. Then, login and navigate to the UPLOAD tab, where you will find detailed instructions on how to use the tool. If you encounter any issues or have any questions when using PACE, please email us at [PLOSMedicine@plos.org](mailto:PLOSMedicine@plos.org).

Response: All figures have been revised based on your requirements. If you still have any problems, please do not hesitate to contact us. We will try our best to solve them.

3) We ask every co-author listed on the manuscript to fill in a contributing author statement, making sure to declare all competing interests. If any of the co-authors have not filled in the statement, we will remind them to do so when the paper is revised. If all statements are not completed in a timely fashion this could hold up the re-review process. If new competing interests are declared later in the revision process, this may also hold up the submission. Should there be a problem getting one of your co-authors to fill in a statement we will be in contact.  YOU MUST NOT ADD OR REMOVE AUTHORS UNLESS YOU HAVE ALERTED THE EDITOR HANDLING THE MANUSCRIPT TO THE CHANGE AND THEY SPECIFICALLY HAVE AGREED TO IT. You can see our competing interests policy here: http://journals.plos.org/plosmedicine/s/competing-interests.

Response: I have added the Authors' Contributions statement and Competing interests statement into the Declarations section in the main text. I can't find the forms of contributing author statement and Competing interests statement in the link above and the author's guide of PLOS MEDICINE. If you still have any problems, please do not hesitate to contact us. We will try our best to solve them. Have a nice day. Best regards.
