# Supplementary material for: Risk factor analysis and creation of an externally-validated prediction model for perioperative stroke following non-cardiac surgery: A multi-center retrospective and modeling study
Source: PLoS Med. 2025 Mar 21;22(3):e1004539. doi: 10.1371/journal.pmed.1004539 (PMC11927879; doi:10.1371/journal.pmed.1004539)
Supplement: Attachment — Submitted filename: Response_to_reviewers_auresp_3.docx [file pmed.1004539.s013.docx]

Thank you for your detailed response to the editors' and reviewers' comments. I have discussed the paper with my colleagues and the academic editor, and it has also been seen again by two of the original reviewers. The changes made to the paper were mostly satisfactory to the reviewers. However, the editorial team concurs with reviewer #4 that their comments regarding the inclusion of perioperative aspirin have not been sufficiently addressed. Therefore, we ask you to carefully address the comments in a further revision and suggest that you provide the data collection forms, which should indicate that you were able to differentiate between pre- and post-operative aspirin, to preclude the need for further revisions and satisfy the reviewers and editors. When submitting your revised paper, please again include a detailed point-by-point response to the comments.

Response: Thanks very much for your work in reviewing our manuscript again. We have carefully addressed the comments from the reviewers. In response to the question raised by reviewer #4 regarding perioperative aspirin as a predictive factor, we expressed absolute recognition and provided a detailed response. As reviewer #4 stated below : “This risk would not exist if the authors were able to include in their model only aspirin use that preceded the day of occurrence of stroke - which is a very improbable thing that they could do, since rarely administrative databases allow to account for this type of precise temporal relationships”, we are currently unable to provide this distinguishing data, which requires a significant amount of work. We added this as a major limitation in the Discussion section. We sincerely hope that these responses and modifications would be recognized by the reviewers and editors.

Comments from the reviewers:

Reviewer #2: We thank the authors for largely addressing our previous concerns; it appears that the exclusions were already included in Supplementary Figure 1 in the original submission - apologies for the oversight.
Response: Thanks very much for your positive comments.

A couple of comments remain:

1. A minor suggestion would be to again standardize the decimal points for percentages in Table 2, if possible.

Response: We thank the reviewer for pointing out this issue, and we have standardized the decimal points for percentages in Table 2.

2. It is stated that "We have stated the reason of not comparising the CHA2DSVASc, ACSNSQIP and MICA were stated in the Discussion section in page 19". However, it is unclear if CHA2DSVASc was discussed (in the paragraph from Line 379 to 385). This might be clarified.

Response: We apologize for the omission in the revision. We have stated the reason of not comparising the CHA2DSVASc in the Discussion section (in the paragraph from Line 384 to 387).

Reviewer #4: It is appreciable how the authors tried to address all the reviewers' comments and modified the manuscript accordingly. The manuscript is overall improved. 
However, I do remain very concerned regarding the inclusion of perioperative aspirin among their predictors (which, not surprisingly, turned out to be among the strongest ones).

My initial comment was: 
"1. Among the variables they consider as possible risk factors, the authors include what they name "perioperative aspirin". The only definition they provide for this is that "the patient received aspirin medication perioperatively." What "perioperatively" mean? If this means (as usually) that they considered when patients took aspirin any time around surgery, inclduing pre-, potentially intra-, and post-operatively (for example on any day after surgery they spent in hospital, or even only on the first few days after surgery), this means this could be aspirin given BECAUSE the patient had an intra- or postoperative stroke. This means, that to include perioperative aspirin as a risk factor or predictor for stroke is reverse association and therefore biased analysis. If this is what they did, the very strong association between perioperative aspirin and stroke shown in the univariable and multivariable models is then easily explained; as explained is the outstanding
discrimination performance (AUC>0.9) the authors found for their model (exceptional for a prognostic model). If this is what they did, the authors should remove this variable from the studied risk factors, and re-run the multivariable analyses, and provide a new model and risk calculator.
If, instead, "perioperative aspirin" had a different meaning (for example, it is preoperative aspirin) this should be amended or clarified."

The authors' response to my comment is:
"Response: We thank the reviewer for this crucial question, which we had not elaborated
clearly in the Methods. We apologize for the confusion caused by our unclear description. "The patient received aspirin medication perioperatively" means that the patients took aspirin both pre-operatively and post-operatively for anti-coagulation medication, not because the patient had an intra- or postoperative stroke. We have clarified this variable in the revised Methods in page 8 in line 161-162."
They then addressed this point is "Perioperative aspirin indicated the patients who took aspirin both pre-operatively and post-operatively for anti-coagulation medication."

Beyond including an erroneous statement (aspirin is not an anticoagulant), this revision does not address the fundamentals of my concern.
The fact that the patient was already on aspirin preoperatively and they were restarted on their aspirin postoperatively, does not change the concern that the choice of restarting aspirin could have been affected by the occurrence of stroke, and the risk of reverse causation still exists. This risk would not exist if the authors were able to include in their model only aspirin use that preceded the day of occurrence of stroke - which is a very improbable thing that they could do, since rarely administrative databases allow to account for this type of precise temporal relationships.

Even if we were to assume that they were able to account for perioperative aspirin use that only preceded the occurrence of stroke, the inclusion of perioperative aspirin in a postoperative stroke prediction model remains both not appropriate and possibly not helpful, as I try to explain with the following points:
- the most probable way here perioperative aspirin is a risk factor (if not for, or in addition to, reverse causation) is being a surrogate (or confounder) for high cardiovascular risk (in addition to the cardiovascular risk predictors they have already included). For example, patients with a history of coronary stenting are those who could have most typically aspirin continued perioperatively, and they could have an increased risk of stroke. To try to have in the model those predictors that in fact explained why aspirin was continued perioperatively would be way more meaningful to a physician reading the article and using the model, than having periop aspirin as a predictor; indeed,
- what could physicians do with this knowledge that, in this observation administrative database, perioperative aspirin continuation was strongly associated with an increased risk of postoperative stroke? When they run this model preoperatively, or even immediately postoperatively, would they decide not to continue aspirin? Even if there could be a biologically potential explanation for a real or even causal relationship between periop aspirin use and stroke (for example, by increasing periop bleeding which could increase stroke), it would NOT be an observational prognostic study to inform this hypothesis. This is not the objective of this study, and it is not an objective of prediction/prognostic studies in general, which typically do not include interventions administered during the time the cohort is followed (which are different from baseline medications) among predictors. 

I remain firm in my suggestion that perioperative aspirin should not be part of this model.

If authors wish not to take this suggestion, I recommend that they justify their choice and address this major limitation in the discussion.

Response: Thank you very much for your detailed explanation, which made us feel enlightened and made us deeply understand the difference between risk factor and predictive factor in the process of constructing a predictive model. We also thank the reviewer for pointing out the erroneous statement of aspirin medication, and we have corrected it in the Methods section. In this cohort study from a retrospective database, we found a strong correlation between perioperative aspirin medication and perioperative stroke occurrence. We are aware of the concern that the variable “perioperative aspirin” was not clearly identified in the current database. Considering the fact that this variable is highly recognizable in clinical practice, we chose to include it as a predictive factor in the model. But we had not precisely identified the temporal relationships between perioperative aspirin medication and the occurrence of perioperative stroke, which may be a reverse association. However, this identification is very difficult and this issue is a major limitation of our study. We have added this major limitation in the Discussion section, hoping to gain your recognition, and we will specifically focus on this issue in future prospective studies to optimize our prediction model.
