# Supplementary material for: Risk factor analysis and creation of an externally-validated prediction model for perioperative stroke following non-cardiac surgery: A multi-center retrospective and modeling study
Source: PLoS Med. 2025 Mar 21;22(3):e1004539. doi: 10.1371/journal.pmed.1004539 (PMC11927879; doi:10.1371/journal.pmed.1004539)
Supplement: Attachment — Submitted filename: Response to comments.docx [file pmed.1004539.s014.docx]

As you will see, the reviewer is still concerned about the inclusion of perioperative aspirin as a risk factor in the model, which the editorial team agrees with. Therefore, after discussing the paper with the editorial team and in line with Reviewer #4's comments, we ask you to include a sensitivity analysis excluding perioperative aspirin from the model and comparing the results with the full model. We strongly feel that it is not sufficient to address the risk factor "perioperative aspirin" as a major limitation in the discussion. Please note that this is a requirement for further consideration of your manuscript at PLOS Medicine.

Response: Thanks very much for your and the reviewer #4’s suggestion, which has greatly benefited us. After careful consideration, we have decided to remove the “perioperative aspirin” indicator from the prediction model. We reconstructed the prediction model and conducted all the work.

Comments from the reviewers: 

Reviewer #4: The authors seem to have appreciated the reasoning around the very important limitations of including perioperative aspirin in their model. However, given the choice, they preferred not to rerun their study without perioperative aspirin in the model, and to discuss the inclusion of perioperative aspirin among the limitations of their study. Their choice is definitely disappointing since the inclusion of aspirin increases (I would say, not surprisingly) the AUC of their model, but dramatically reduces the ability to interpret what their model is for, and its utility and application in clinical practice when we are called to assess the risk of perioperative stroke of patients undergoing noncardiac surgery. This is a large database, to have a properly done model would have been great. "Considering the fact that this variable is highly recognizable in clinical practice" is not a sound justification to include it as a predictive factor, neither methodologically (as
indicated in my previous comments) not practically, since, if the variable in the model stands for use of aspirin before and after surgery, the model can be applied only after surgery - and when should we calculate this predictor for our patient? How many days after surgery should we wait to decide whether this is a "yes aspirin" or a "no aspirin"? would a stroke have already occurred at that point?. This is to say that, if the model stays as it is, with perioperative aspirin included, authors should mitigate any claim in their paper for using the model to improve clinicians' ability to predict the risk of perioperative stroke. Theirs is a multivariable analysis for perioperative factors associated with the occurrence of perioperative stroke (including possibly after-the-fact variables). 

I would also suggest two additional revisions around the same issue:
- in the Methods, I would further clarify what is perioperative use of aspirin. Currently they say "the situation of aspirin medication both pre-operatively and post-operatively". Was this a binary variable? If so, what patients did the "no" category include?  Did it include both those who were not on aspirin at baseline, and those who were on aspirin at baseline but did not continue it after surgery?
- in the Discussion of this as a study limitation, the authors added "Thirdly, due to the deficiency of retrospective database, the precise temporal relationship between perioperative aspirin medication and the occurrence of perioperative stroke was difficult to identify, which may lead to a biased analysis owing to the potential reverse association between this variable and the outcome. In addition, as an intervention administered during the perioperative period, aspirin medication could be a surrogate for high stroke risk, and the reason why aspirin was continued perioperatively should be taken into consideration during clinical application of this prediction model as it would not change clinical medication decision in these high risk patients."  I suggest to rephrase it as "Thirdly, we found that perioperative aspirin was strongly associated with perioperative cover stroke. This could have been due to aspirin being a marker of a high cardiovascular risk that other
variables included in the model did not fully capture; however, this could also be due reverse association. In fact, due to the retrospective nature of our analysis, based on an administrative database, we could not determine the precise temporal relationship between perioperative aspirin use and the occurrence of perioperative stroke. This represents a major limitation to the application of our model for the prediction of the risk of perioperative stroke in clinical routine."

Response: Thanks very much for your suggestion and persistance. After careful consideration, we believe that your suggestion is completely correct, which would make our prediction model more clinically meaningful. Therefore, we have decided to remove the “perioperative aspirin” indicator from the prediction model. We have reconstructed the prediction model and conducted all the work.
