# Supplementary material for: Risk factor analysis and creation of an externally-validated prediction model for perioperative stroke following non-cardiac surgery: A multi-center retrospective and modeling study
Source: PLoS Med. 2025 Mar 21;22(3):e1004539. doi: 10.1371/journal.pmed.1004539 (PMC11927879; doi:10.1371/journal.pmed.1004539)
Supplement: Attachment — Submitted filename: Respnse to Comments.docx [file pmed.1004539.s015.docx]

Comments from Reviewers:

Reviewer #2: We thank the authors for addressing our previous comments.

Reviewer #4: My thanks and congratulations to the authors for the efforts they made to change their analysis and address the reviewer's and editors' concerns. Their work is now more solid and has a more straightforward interpretation and potential for use in clinical practice.

A few minor suggestions:

1) The authors can now remove the definition of perioperative aspirin and its inclusion (table 1) among the patient characteristics since this is no longer a variable of relevance in their paper.

Response: Thanks very much for reviewing our manuscript again. We have removed the definition of perioperative aspirin and its inclusion (both table 1 and Supplementary Table 3) among the patient characteristics.

2) The authors should clarify the statement regarding the DCA: "In the current model, the NB was 0.002 if all the subjects were treated, and the NB was 0 if none of them were treated." "Treated" with what? Do they mean "if the model was used"?

Response: We thank the reviewer for pointing out this issue, and we have illustrated in the manuscript that“treated” refers to patients receiving interventions based on the prediction model..

3) When they decided to translate the model into categories of risk, why dod they decide to have only one threshold and only two categories, i.e., low and high risk? To have at least 3 categories may be more informative. In any case, I believe that the average absolute risk estimate for each category is what has most clinical relevance. For this reason, in the two examples that they include in the model application section they should provide the corresponding estimated absolute risk rather than saying that their score positioned them into the low vs high risk category.

Response: We thank the reviewer for this inspiring suggestion, and we agree with the viewpoint that at least 3 categories may be more informative. Considering that 3 categories may not necessarily change the actual clinical decision and may increase intervention complexity during application, we choose to retain the current two categories. We have provided the corresponding estimated absolute risk instead of the score in the manuscript.

4) Page 16, lines 333-334: "which was similar to two recent comprehensive studies" should read as "which was similar to the incidence found in two recent comprehensive studies".

Response: We thank the reviewer for pointing out this issue, and we have corrected the sentence "which was similar to two recent comprehensive studies" as suggested.

Any attachments provided with reviews can be seen via the following link:

------------------------------------------------------------

Academic Editor Comments:

On the aspirin question - the authors have taken it out of the model but still include it in their list of data collected. This is entirely appropriate, but the authors need to either:

1. Explain in the methods why they subsequently did not use the aspirin data in their model (e.g. by paraphrasing reviewer 4's discussion), or

2. Provide the aspirin results (in a supplementary section if necessary) and then explain in the discussion why they did not use them (as above).

------------------------------------------------------------

Response: We thank the Editor for pointing out this issue. Since perioperative aspirin is no longer a variable of relevance in the paper, we have removed the definition of perioperative aspirin and its inclusion (both table 1 and Supplementary Table 3) among the patient characteristics, as suggested by Reviewer #4.

DATA AVAILABILITY

Please note that the data availability statement in the online submission form and in the main text on lines 418-419 do not match. Please revise according to the following:

a) If the data are freely or publicly available, note this and state the location of the data: within the paper, in Supporting Information files, or in a public repository (include the DOI or accession number).

b) If the data are owned by a third party but freely available upon request, please note this and state the owner of the data set and contact information for data requests (web or email address). Note that a study author cannot be the contact person for the data.

c) If the data are not freely available, please describe briefly the ethical, legal, or contractual restriction that prevents you from sharing it. Please also include an appropriate contact (web or email address) for inquiries (again, this cannot be a study author).

CODE AVAILABILITY

We expect all researchers with submissions to PLOS in which author-generated codeunderpins the findings in the manuscript to make all author-generated code available without restrictions upon publication of the work. In cases where code is central to the manuscript, we may require the code to be made available as a condition of publication. Authors are responsible for ensuring that the code is reusable and well documented. Please make any custom code available, either as part of your data deposition or as a supplementary file. Please add a sentence to your data availability statement regarding any code used in the study, e.g. "The code used in the analysis is available from Github [URL] and archived in Zenodo [DOI link]" Please review our guidelines at https://journals.plos.org/plosmedicine/s/materials-software-and-code-sharing and ensure that your code is shared in a way that follows best practice and facilitates reproducibility and reuse. Because Github depositions can be

readily changed or deleted, we encourage you to make a permanent DOI'd copy (e.g. in Zenodo) and provide the URL.

TITLE

Please revise your title according to PLOS Medicine's style. Your title must be nondeclarative and not a question. It should begin with main concept if possible. "Effect of" should be used only if causality can be inferred, i.e., for an RCT. Please place the study design ("A randomized controlled trial," "A retrospective study," "A modelling study," etc.) in the subtitle (ie, after a colon).

我们的题目应该符合要求。

ABSTRACT

1) l.7: We suggest clarifying that the model was built to predict the incidence of perioperative stroke after non-cardiac surgery.

Response: We thank the Editor for this suggestion, and we have clarified in the manuscript that the model was built to predict the incidence of perioperative stroke after non-cardiac surgery.

2) l.11ff: PLOS Medicine prefers the use of patient-centered language, e.g. patients undergoing non-cardiac surgery (or similar). Please revise throughout the manuscript, including tables and figures (including those in the Supporting Information).

Response: We thank the Editor for pointing out this issue, and we have revised the expression throughout the manuscript.

3) l.13: We suggest briefly mentioning the method used to narrow the risk factors down to the thirteen included. For example: After multivariate analysis and stepwise elimination (P<0.05), thirteen variables…” (or similar).

Response: We thank the Editor for this suggestion, and we have modified the Abstract section as suggested.

4) l.14: Please define ‘ASA’.

Response: We thank the Editor for pointing out this issue, and we have defined ‘ASA’in the Abstract.

5) l.19ff: Please report statistical information as follows to improve clarity for the reader "22% (95% CI [13%,28%]; p</=). For example: “0.893 (95% confidence interval (CI) [0.879,0.908]; P<0.001)”. When reporting 95% Cis, please separate upper and lower bounds with commas instead of hyphens as the latter can be confused with reporting of negative values. Please revise throughout the manuscript.

Response: We thank the Editor for pointing out this issue, and we have revised the 95% Cis throughout the manuscript, including the tables and figures.

6) ll.31-35: We suggest splitting the sentence into two sentences: In this work, we identified thirteen independent risk factors for perioperative stroke and constructed an effective prediction model with well-supported external validation in Chinese patients undergoing surgery. The model may provide potential intervention targets and help to screen high-risk patients for perioperative stroke prevention.

Response: We thank the Editor for this suggestion, and we have split the sentence as suggested.

7) Please ensure that all numbers presented in the abstract are present and identical to numbers presented in the main manuscript text.

Response: We have checked the manuscript to make sure that all numbers presented in the abstract are present and identical to numbers presented in the main manuscript text.

AUTHOR SUMMARY

1) ll.50-54: Please define all abbreviations used for the first time (ASA, MAP, FAR, FPG).

Response: We thank the Editor for pointing out this issue, and we have defined all abbreviations used for the first time (ASA, MAP, FAR, FPG).

2) ll.61-62: Please revise and clarify what you mean by providing potential intervention targets.

Response: We thank the Editor for pointing out this issue, and we have clarified the specific potential intervention targets in the manuscript.

INTRODUCTION

If there has been a systematic review of the evidence related to your study (or you have conducted one), please refer to and reference that review and indicate whether it supports the need for your study.

Response: Thanks very much for your suggestion. We have included a systematic review (Reference 13) in the Introduction, which supports the need for our study. We stated as follows: “However, in a recent authoritative systemic review, the author cautioned the use of this prediction model because it has not been validated externally [13].”

METHODS AND RESULTS

1) l.153: Please define ‘ICD’ at first use.

Response: We thank the Editor for pointing out this issue, and we have defined ‘ICD’in the Methods section.

2) l.224: Please replace ‘exhibited’ with ‘experienced’.

Response: We thank the Editor for this advice, and we have replaced ‘exhibited’ with ‘experienced’.

3) ll.251-252: “As analyses were adjusted in the final model…” – adjusted for? Please specify and ensure that these adjustments are described in the Methods section.

Response: We thank the Editor for pointing out this issue. Stepwise elimination was performed with P<0.05 for retaining a given predictor in the model. But for preoperative β-blockers (P=0.093) and preoperative steroids (P=0.057), we included the two variables in the final model, considering the clinical significance. We have described the adjustments in the Model development and validation part in the Methods section.

4) ll.285-286, please revise: “The model exhibited a well-calibrated performance in the cohorts from Nanfang Hospital (Figure 2C) and Henan Provincial People's Hospital (Figure 2D),…”

Response: We thank the Editor for this advice, and we have revised the sentence as suggested.

5) Figure 1/2: Please provide definitions of numerical values (in parentheses). Please define 'Pr'.

Response: We thank the Editor for pointing out these issues. We have defined the numerical values in parentheses in Figure 1 and Figure 2. We have also replaced 'Pr' with ‘probability’ in Figure 1 and Figure 2 to make the figures easier to understand.

6) Table 1: Please remove the p-values from Table 1. Please define ‘yr’ or write ‘years’.

Response: We thank the Editor for this advice. We have removed the p-values from Table 1 and have replaced ‘yr’ with ‘years’. Furthermore, we have also removed the p-values from Supplementary Table 3.

DISCUSSION

General guidance: Please present and organize the Discussion as follows: a short, clear summary of the article's findings; what the study adds to existing research and where and why the results may differ from previous research; strengths and limitations of the study; implications and next steps for research, clinical practice, and/or public policy; one-paragraph conclusion.

1) l.326/341/343/345/347/387: Please temper claims of primacy of results by stating, "to our knowledge" or something similar.

Response: We have tempered claims of primacy of results in the revision by stating “to our knowledge”, “identified” and “in our opinion” .

2) l.355/357: Please provide references.

Response: We have added a reference.

3) Please remove any subheadings. The conclusion should be a continuous part of the discussion.

Response: We have removed the subheading “Conclusion”.

SUPPLEMENTARY MATERIAL

In the published article, supporting information files are accessed only through a hyperlink attached to the captions. For this reason, you must list captions at the end of your manuscript file. You may include a caption within the supporting information file itself, as long as that caption is also provided in the manuscript file. Do not submit a separate caption file.

When SI files are contained with a single file:

Please label the file as ‘S1 Supporting Information’.

Please apply alphabetical labelling to each table and figure contained within the S1 file. For example, ‘Fig A’ to ‘Fig Z’ and ‘Table A’ to ‘Table Z’.

Plain text does not need to be labelled and can just be given a title as necessary. For example, ‘Statistical Analysis Plan’.

Please cite tables/figures as ‘Fig A in S1 Supporting Information’ and/or ‘Table A in S1 Supporting Information’, for example.

Please cite plain text as, ‘Statistical Analysis Plan in S1 Supporting Information’, for example.

When SI files are uploaded as separate files:

Please label tables as ‘S1 Table’ (so on) and figures as ‘S1 Fig’ (and so on).

Any additional documents (protocols/analysis plans etc.) can be labelled as ‘S1 Protocol’, for example. Please cite items as exactly as labelled.

Response: We have edited them based on your comments. If you still have any problem, please do not hesitate to contact us. We will try our best to solve them.

------------------------------------------------------------

General Editorial Requests

1) We ask every co-author listed on the manuscript to fill in a contributing author statement. If any of the co-authors have not filled in the statement, we will remind them to do so when the paper is revised. If all statements are not completed in a timely fashion this could hold up the re-review process. Should there be a problem getting one of your co-authors to fill in a statement we will be in contact. YOU MUST NOT ADD OR REMOVE AUTHORS UNLESS YOU HAVE ALERTED THE EDITOR HANDLING THE MANUSCRIPT TO THE CHANGE AND THEY SPECIFICALLY HAVE AGREED TO IT.

Response: We have added Authors' Contributions statement in the revised manuscript. If you still have any problem, please do not hesitate to contact us. We will try our best to solve them.

2) Please ensure that the paper adheres to the PLOS Data Availability Policy (see http://journals.plos.org/plosmedicine/s/data-availability), which requires that all data underlying the study's findings be provided in a repository or as Supporting Information. For data residing with a third party, authors are required to provide instructions with contact information for obtaining the data. PLOS journals do not allow statements supported by "data not shown" or "unpublished results." For such statements, authors must provide supporting data or cite public sources that include it.

Response: We have added Availability of data and materials in the revised manuscript. If you still have any problem, please do not hesitate to contact us. We will try our best to solve them.

3) Please review your reference list to ensure that it is complete and correct. If you have cited papers that have been retracted, please include the rationale for doing so in the manuscript text, or remove these references and replace them with relevant current references. Any changes to the reference list should be mentioned in the rebuttal letter that accompanies your revised manuscript.

Response: We have checked the reference list in the revised manuscript. If you still have any problem, please do not hesitate to contact us. We will try our best to solve them.
